# ID3 contributes to cerebrospinal fluid seeding and poor prognosis in medulloblastoma

**DOI:** 10.1186/1471-2407-13-291

**Published:** 2013-06-15

**Authors:** Ji Hoon Phi, Seung Ah Choi, Sang-Hee Lim, Joongyub Lee, Kyu-Chang Wang, Sung-Hye Park, Seung-Ki Kim

**Affiliations:** 1 Division of Pediatric Neurosurgery, Seoul National University Children’s Hospital, Seoul, Republic of Korea; 2 Adolescent Cancer Center, Seoul National University Cancer Hospital, Seoul, Republic of Korea; 3 Medical Research Collaborating Center, Seoul National University Hospital, Seoul, Republic of Korea; 4 Department of Pathology, Seoul National University Children’s Hospital, Seoul, Republic of Korea

**Keywords:** ID3, Medulloblastoma, Seeding, Prognosis, Survival, Group 4

## Abstract

**Background:**

The inhibitor of differentiation (ID) genes have been implicated as promoters of tumor progression and metastasis in many human cancers. The current study investigated the expression and functional roles of ID genes in seeding and prognosis of medulloblastoma.

**Methods:**

ID gene expression was screened in human medulloblastoma tissues. Knockdown of ID3 gene was performed in medulloblastoma cells in vitro. The expression of metastasis-related genes after ID3 knockdown was assessed. The effect of ID3 knockdown on tumor seeding was observed in an animal model in vivo. The survival of medulloblastoma patients was plotted according to the ID3 expression levels.

**Results:**

Significantly higher ID3 expression was observed in medulloblastoma with cerebrospinal fluid seeding than tumors without seeding. Knockdown of ID3 decreased proliferation, increased apoptosis, and suppressed the migration of D283 medulloblastoma cells in vitro. In a seeding model of medulloblastoma, ID3 knockdown in vivo with shRNA inhibited the growth of primary tumors, prevented the development of leptomeningeal seeding, and prolonged animal survival. High ID3 expression was associated with shorter survival of medulloblastoma patients, especially in Group 4 medulloblastomas.

**Conclusions:**

High ID3 expression is associated with medullolbastoma seeding and is a poor prognostic factor, especially in patients with Group 4 tumors. ID3 may represent the metastatic/ aggressive phenotype of a subgroup of medulloblastoma.

## Background

Medulloblastoma is an aggressive neoplasm developing in the cerebellum of children. Long-term survival rates of children with medulloblastoma have increased since 1980s with adoption of whole neuraxis irradiation and chemotherapy [1,2]. However, a substantial portion of patients still have a grim prognosis despite intensified therapies. Poor prognostic factors of newly diagnosed medulloblastomas are well known in large clinical trials: a young age of onset (< 3 yrs), a large residual tumor after surgery, tumor dissemination (seeding) into the cerebrospinal fluid (CSF), and possibly an anaplastic/large cell histology [3-5]. Among these clinical factors, tumor seeding at presentation may have the strongest impact on patient prognosis, as described in many studies [6,7].

Our previous study on medulloblastoma demonstrated that patients with tumor seeding at presentation had a 5-year survival rate of 38% in contrast to 73% for patients without tumor seeding [8]. Although both medulloblastoma and glioma are intra-axial tumors, their patterns of dissemination are quite different. Medulloblastoma frequently ‘seeds’ through the CSF pathway into spinal and intracranial subarachnoid spaces, but gliomas usually infiltrate white matter tracts that are adjacent to the primary tumor [9,10]. Large-scale genomic analyses revealed the multiple origins and molecular pathogenesis of medulloblastoma [11-14]. Recently, several studies have been focused on the mechanism of medulloblastoma seeding because better understanding of the phenomenon may lead to dramatic therapeutic improvement. Researchers in Toronto revealed that metastatic cells of medulloblastoma have distinct genetic variations and identified some candidate genes related to medulloblastoma seeding through functional genomics [15,16]. Furthermore, downstream targets of MYC oncogene and tumor-promoting microRNAs have also been implicated as drivers of medulloblastoma dissemination [17,18]. However, as medulloblastoma has diverse pathogenetic origins, many different genes may function as key metastasis-promoting genes in subgroups of patients. Therefore, it may be important to search for candidate genes using human medulloblastoma tissues.

Inhibitor of differentiation (ID) genes encode transcription factors with a basic helix-loop-helix (bHLH) motif that act as suppressors of cellular differentiation [19,20]. ID molecules are involved in a wide range of cellular processes such as cell proliferation and migration. Interestingly, ID genes are overexpressed in many human cancers of epithelial origin, such as esophageal, pancreatic, colorectal, prostate, and breast cancer [21-25]. ID genes promote tumor cell migration, invasion, and angiogenesis which are key components of tumor metastasis [19,26]. Therefore, ID genes are potential metastasis-promoting genes that confer aggressiveness to epithelial tumors. Therefore, ID genes could be candidate genes for human medulloblastoma seeding.

This study investigated the expression of ID genes in human medulloblastoma and demonstrated that ID3 overexpression was significantly associated with tumor seeding and poor prognosis of the patients. In vitro and in vivo studies demonstrated that the ID3 gene participated in suppression of apoptosis and the migration of medulloblastoma cells. Interestingly, a subgroup of medulloblastoma, Group 4 tumors showed significantly higher ID3 expression than the other subgroups. High ID3 expression was a poor prognostic factor, especially in patients with Group 4 tumors. ID3 may represent the metastatic/ aggressive phenotype of a subgroup of medulloblastoma.

## Methods

### Tumor tissues and cell lines

All studies reported here were performed with approval of the Institutional Review Board of the Seoul National University Hospital. Snap-frozen medulloblastoma tissue from 39 patients was retrieved from the Brain Bank of the Division of Pediatric Neurosurgery, Seoul National University Children’s Hospital. Normal cerebellar tissue was retrieved from the same tissue bank for use as a control. Patient selection was based on the availability of snap-frozen tissues. The person who selected the patients was blind to patient’s clinical information except diagnosis. Human medulloblastoma cell lines (D283 and Daoy) were purchased from the American Type Culture Collection (ATCC, Manassas, VA). D283 cells were cultured in Minimum Essential Medium Eagle (EMEM; ATCC), and Daoy cells were cultured in Dulbecco’s Modified Eagle’s Medium (DMEM; Invitrogen, Carlsbad, CA) supplemented with 10% (v/v) fetal bovine serum (FBS; Invitrogen) and penicillin-streptomycin (1× final concentration; Invitrogen). All cells were incubated at 37°C in a 5% CO_2_/ 95% air atmosphere.

### Real-time quantitative polymerase chain reaction (PCR)

The levels of mRNA transcription were assessed by real-time quantitative PCR (RT-qPCR) using TaqMan probes (Applied Biosystems, Carlsbad, CA) in an ABI 7000 system. TaqMan probes for ID1 (ABI Catalog number Hs00357821), ID2 (Hs00747379), ID3 (Hs00171409), ID4 (Hs00155465), and glyceraldehyde 3-phosphate dehydrogenase (GAPDH; Hs99999905) were used. The reactions were performed under the conditions specified in the ABI TaqMan Gene Quantitation assay protocol, and all reactions were repeated in triplets. The comparative threshold cycle (ΔCt) method calculated the relative gene expression, normalized to GAPDH and relative to normal brain expression [27].

### siRNA and shRNA knockdown of ID3

siRNA and shRNA were used to knock down ID3 expression in the D283 cell line. Control siRNA (#SN1002) and ID3-siRNA (#1072358) were designed and synthesized by Bioneer (Daejeon, Korea) for the in vitro studies. Transfection of the control-siRNA and ID3-siRNA was performed using Lipofectamine RNAiMax (Invitrogen) following the manufacturer’s instructions. Lentiviral particles containing shRNA targeting the human ID3 (SHCHLNG-NM_002167), nontargeting shRNA (SHC002V), and GFP (SHC003V)-containing control transduction particles were obtained from Sigma-Aldrich (St. Louis, MO) for in vivo studies. D283 cells were seeded in 96-well plates and transduced in 110 μl of EMEM containing 10% FBS and 8 μg/ml hexadimethrine bromide. The cells were reseeded in 6-well plates 24 hrs after incubation and selected using 1 μg/ml puromycin for 7 days. Knockdown efficiency and specificity with siRNA and shRNA was confirmed using RT-qPCR with the gene expression normalized to GAPDH. Knockdown of ID gene expression was further confirmed by western blot.

### ID3 rescue experiment

To prove the specificity of the ID3-shRNA knockdown, full-length ID3 cDNA (360 bp) was synthesized using the RT–PCR Kit (Clontech, Mountain View, CA) from RNA extraction of D283 cells. Constructs were inserted into the BamH1/Xho1cloning site of pEGFP.C2 (BD Biosciences, San Jose, CA) and then transfected into the ID3 knockdown D283 cell line using the Neon® Transfection (Invitrogen) according to the manufacturer’s instructions with some modifications. ID3-shRNA knockdown cells (1 × 10^7^) were resuspended in 120 μl of Neon® Resuspension Buffer R with 12 μg of plasmid DNA pulsed once according to the manufacturer’s instructions. After the pulse, cells were quickly transferred into EMEM media containing 10% FBS. Cells transfected with a pEGFP.C2 vector were used as a control. Expression of green fluorescent protein (GFP) was observed by fluorescence microscopy 24 hrs after nucleofection. The cells were then incubated for 48 hrs before RNA and protein collection for further experiments.

### Western blot

After Transfection with siRNA negative control or ID3-siRNA, cells were resuspended in protein extraction solution (Intron Biotech, Seongnam, Korea), according to the manufacturer’s protocol. Protein concentrations were determined using BCA protein assay kit (Thermo Fisher Scientific, Waltham, MA). For immunoblottg, 40 μg of protein was prepared in SDS sample buffer (Invitrogen), boiled for 10 min at 70°C and electrophoresed on a 4–12% gradient bis-Tris gel (Invitrogen). The proteins were then electotransfered to a polyvinylidene fluoride membrane (Invitrogen) using iblot transfer system (Invitrogen). After the membrane had been blocked with Tris-buffered saline (TBS) containing 5% nonfat dry milk, it was incubated overnight at 4°C with the following anti-ID1 antibody (1:500; Abnova, Taipei, Taiwan), anti-ID2 antibody (1:500; Cell Signaling Technology, Danvers, MA), anti-ID3 antibody (1:500; Cell Signaling Technology), anti-ID4 antibody (1:250; Abcam, Cambridge, UK) and monoclonal anti-β-actin antibody (1:10,000; Sigma-Aldrich) in TBS containing 0.1% Tween-20. After the blot was washed, it was incubated with horseradish-peroxidase–conjugated species-specific secondary antibody (1:5,000; Jackson Laboratory, West Grove, PA) for 1 hr at room temperature. After the blots had been washed several times in TBS with 0.1% Tween-20, they were developed with enhanced chemiluminescence reagent (Invitrogen) and exposed to Kodak BioMax autoradiography film, and developed.

### Viability assay

D283 cells were transfected with control-siRNA or ID3-siRNA, seeded in 96-well plates (5 × 10^3^), and incubated for 48 hrs. CCK (cell counting kit; Dojindo, Kumamoto, Japan) was added and incubated for 2 hrs. Then, absorbance of each well was measured at 540 nm using a micro-ELISA reader (Molecular Devices, Sunnyvale, CA). The percentage of cellular survival was determined using the relative absorbance of ID3-siRNA-transfected cells versus control-siRNA-transfected cells. All in vitro assays were performed in triplicate.

### Proliferation assay

The proliferation rates of D283 cells were measured using a BrdU ELISA kit (Roche Diagnostics, Basel, Switzerland) 48 hrs after transfection with control- or ID3-siRNA. The cells were plated in 96-well plates at an equal density (5 × 10^3^). BrdU was added to the cells for 4 hrs, and the cells were treated according to the manufacture’s protocol. The optical density at 450 nm was measured using an ELISA plate reader.

### Apoptosis assay

TUNEL assay was performed for the detection of apoptotic cells using an ApopTag Peroxidase In situ Apoptosis Detection Kit (Chemicon, Temecula, CA). D283 cells were transfected with control- or ID3-siRNA (1 × 10^4^) and cultured in 2-well chamber slides for 24-48 hrs. The cells were fixed and stained according to the manufacturer’s instructions. Apoptotic cells were observed and quantified in 5 randomly chosen high-power fields under a light microscope. The apoptosis index was defined as a percentage of the observed apoptotic cells in 1,000 cells.

### Cellular senescence assay

Senescence-associated ß-galactosidase (SA-ß-gal) activity was detected using the Cellular Senescence Assay Kit (Chemicon), according to the manufacturer’s instructions. D283 cells were transfected with control- or ID3-siRNA (1 × 10^4^), seeded in 6-well plates, and incubated for 16 hrs at 37°C. Representative microscopic fields were photographed under a 10× objective lens.

### Cell cycle analysis

D283 cells were transfected with control- or ID3-siRNA (1 × 10^6^) and detached by scraping. The cells were fixed in 70% iced-cold ethanol with vortexing and incubation for 1 hr at 20°C. The cells were washed with cold PBS and resuspended with 0.5 mg/ml Rnase A (Sigma-Aldrich). After 1 hr at 37°C, 10 μg/ml propidium iodine solution (Sigma-Aldrich) was added in the dark at 4°C and the cells were observed with fluorescent microscopy. The cells were analyzed using fluorescence-activated cell sorting (FACS).

### Migration assay

D283 cells were transfected with control- or ID3-siRNA prior to seeding onto the upper chamber of a Transwell (8 μm pore size; Corning Costar, Corning, NY). The cells (5 × 10^4^/ well) were harvested after transfection and introduced into the upper chamber. The cells in the upper chamber were maintained in serum-free medium that included mitomycin-C (10 μg/ml; Sigma-Aldrich), and the lower chamber was filled with culture medium supplemented with 10% fetal bovine serum as the chemoattractant. The cells without siRNA treatment were included as reagent control. The remaining cells at the upper surface were completely removed using a cotton swab after 16 hrs. The migrated cells on the lower surfaces of the membranes were fixed and stained in a solution of 1% (w/v) crystal violet in 2% ethanol for 30 seconds and rinsed in distilled water. The migrated cells were quantified in 5 randomly chosen fields. The assays were performed in triplicate.

### mRNA miniarray for 94 genes related to cellular invasion and migration

The mRNA expression of 94 cellular invasion and migration gene was analyzed using a ready-to-use Array Human Extracellular Matrix & Adhesion Molecules 96-well Plate (Applied Biosystems; Catalog number, 4414133) and the ABI 7500 Real-Time qPCR system. Selected genes that demonstrated large discrepancies were confirmed using RT-PCR. The primer sequences and PCR parameters are summarized in Additional file 1: Table S1.

### Reverse transcriptase polymerase chain reaction

Total RNA was isolated from human tissues and tumor cell lines using a PureLink RNA mini-kit (Invitrogen). cDNA synthesis was performed using EcoDry Premix-Random hexamers (Clontech, Madison, WI), following the manufacturer’s instructions. PCR amplification was performed using AccuPower PCR premix (Bioneer). The primer sequences and PCR parameters are summarized in Additional file 1: Table S1. The PCR products were resolved on a 1% agarose gel stained with ethidium bromide and visualized using a UV transilluminator.

### Immunohistochemistry

Four paraffin-embedded medulloblastoma tissues (2 tissues from patients with seeding and 2 tissues from patients without seeding) were sectioned at 4 μm using a microtome and transferred to silane-coated slides. Immunohistochemistry was performed as described previously [28]. Primary antibodies and their concentrations were applied as follows: ID3 (1:50; Spring Bioscience, Pleasanton, CA), tissue inhibitor of metalloproteinase 3 (TIMP3; 1:100; Novus Biologicals, Littleton, CO), integrin beta 4 (ITGB4; 1:50; Abcam), collagen type XII alpha1 (COL12A1; 1:1; Abnova), ADAM metallopeptidase with thrombospondin type 1 motif 8 (ADAMTS8; 1:100; Abbiotec, San Diego, CA), tenascin C (TNC; 1:100; Abcam), connective tissue growth factor (CTGF; 1:600; Abcam), and intercellular adhesion molecule 1 (ICAM1; 1:25; Cell Signaling Technology).

### Animal model and inhibition of tumor seeding in vivo

The Institutional Animal Care and Use Committee of Seoul National University College of Medicine approved all animal experiment protocols. Transplantation of cells into female BALB/cnude mice was performed under aseptic conditions. D283 cells were labeled using fluorescent magnetic nanoparticle (LEO-LiveTM-675; Biterials, Seoul, Korea) for live in vivo imaging or chloromethylbenzamido-DiI (Molecular Probes, Eugene, OR) for Immunofluorescence staining. The cells were washed three times after a 24-hour incubation and suspended in PBS at a concentration of 1.5 × 10^6^ cells per 30 μl. Mice were anesthetized using an intraperitoneal injection of 100 mg/kg ketamine (Yuhan, Seoul, Korea) and 10 mg/kg xylazine (Bayer Korea, Seoul, Korea). The mouse heads were fixed in a stereotactic guiding device (David Kopf Instruments, Tujunga, CA), and the cisterna magna was exposed under a microscopic view. Labeled cells were slowly injected into the subarachnoid space of the cisterna magna using a 30-gauge needle [29].

Live in vivo image acquisition and analysis were performed using an in vivo multispectral imaging system (CRi Maestro™ In-Vivo Imaging System; Cri, Hopkinton, MA). The injected cells were observed using an in vivo multispectral imaging system every 3–4 days. The regions of interest (ROI) were drawn over the tumor and normal tissue, and the average signal (×10^6^ photons/cm^2^ × s) for each area was measured.

The longitudinal length from the cranial to caudal ends of the signal was measured to evaluate the extent of seeding. The mice were perfused with 4% paraformaldehyde under deep anesthesia and sacrificed 30 days after cellular implantation. Whole brains and spinal cords were fixed and dehydrated in graded sucrose concentrations. The tissues were embedded in OCT compound (Tissue-Tek®; Sakura, Tokyo, Japan) and stored at −80°C. The brains were sectioned sagittally into 10 μm-thick slices using a cryostat. Spinal cords were sectioned in 5-μm intervals beginning at the cervicomedullary junction. The sections were stained with hematoxylin and eosin.

Immunofluorescence staining was further performed on the sections to confirm the presence of proliferating and apoptotic cells. Sectioned tissues were washed and the primary antibodies were applied. Primary antibodies used were anti-ID2 antibody (1:100; Cell Signaling Technology), anti-ID3 antibody (1:200; Cell Signaling Technology), anti-ID4 antibody (1:50; Abcam), and anti-Ki-67 nuclear antigen (1:400; Abcam) for proliferating cell staining, anti-caspase-3 (1:200; Cell Signaling Technology) for apoptotic cell staining, and anti-human nuclei (1:250; Millipore, Billerica, MA). Alexa Fluor 488 or 594-conjugated goat anti-rabbit or mouse immunoglobulin-G (IgG) (1:200; Invitrogen) were used for secondary antibodies. After washing, tissue sections were mounted with an anti-fading solution containing 4′-6-diamidino-2-phenylindole (DAPI; Vector Laboratories, Burlingame, CA) and negative control staining were established by omitting the primary antibodies. The apoptotic and proliferating indices were defined as the percentage of positive nuclei within the total number of nuclei in three random fields. The sections were observed using a confocal microscope (Carl Zeiss, Oberkochen, Germany).

### Review of patient clinical data

The electronic medical records (EMR) of selected patients were reviewed retrospectively. All patients received initial surgical resection at the Seoul National University Children’s Hospital from Nov. 1998 to Apr. 2009. Clinical data included the patient’s age at diagnosis, sex, tumor histology, and tumor seeding at presentation. Patient survival was confirmed from follow-up sheets in the EMR. Tumor progression was defined as radiological documentation of a new tumor or the growth of preexisting masses (> 25% in 2-D analyses). All patients were followed up from the time of initial surgery (initial diagnosis) until the date of death or until Jan 29, 2013, whichever occurred first. Death certificate information was retrieved from the National Statistical Office and the Ministry of Public Administration and Security. A structured data extraction form was developed in which clinical data from the EMR was merged with the death certificate information and experimental data (i.e., the expression levels of specific genes).

### Identification of molecular subgroup of tumors

Medulloblastomas are heterogeneous tumors consisting of at least 4 distinct molecular subgroups [13]. Tumor tissues from 31 patients out of the 39 patients comprising this study were also included in a large-scale genomic study of Dr. Taylor MD, in which subgroup affiliation was performed using a nanoString-based RNA assay [30]. The information of subgroup allocations were obtained for 30 patients and the tumors were divided into WNT, SHH, Group 3, and Group 4 medulloblastoma (information courtesy of Dr. Taylor MD).

### Statistical analyses

A Mann–Whitney U test or Student t test were applied to compare continuous variables between 2 groups. Analysis of variance (ANOVA) was used for comparison of data between 3 groups. Progression-free survival (PFS) was defined as the time interval from the day of initial surgery to the date that tumor progression was documented radiologically or the date of the last follow-up. Overall survival (OS) was defined as the time interval from the day of initial surgery to the date the patient died or the date of the last follow-up. We explored the best cutoff point of ID3 for predicting occurrence of death or progression of MB using receiver operator characteristic (ROC) analyses without considering the length of follow-up. Since the value of 6.007 was optimal for both outcomes, a high expression level of ID3 was defined as a greater than 6.007-fold increase in mRNA expression in RT-qPCR normalized to controls. Survival in each group was analyzed using a Kaplan-Meier method. A log-rank test was used for comparisons of survival data between groups. Multivariate analyses of PFS and OS were conducted using the Cox proportional hazard model. Clinical variables with a P value less than 0.1 in univariate analyses were included in the multivariate models. Basic clinical variables such as sex and age at diagnosis were included in multivariate analyses regardless of their P values. All tests were two-sided, and a P value less than 0.05 was considered significant. MedCalc version 12.4.0 (MedCalc, Ostend, Belgium; a free-trial version) was used for ROC analysis and IBM-SPSS version 19.0 software (IBM, Armonk, NY) was used for all the other statistical analyses.

## Results

### Expression of ID genes in human medulloblastoma

The mRNA expression of ID genes (ID1, ID2, ID3, and ID4) was assessed in human medulloblastoma tissues using RT-qPCR (N = 39 for ID3 and N = 37 for ID1, ID2, and ID4). The average expression levels for ID1, ID2, and ID4 in medulloblastoma were lower than the expression levels in normal cerebellum. There were strong positive correlations between ID1 and ID4 (Pearson coefficient r = 0.381, P = 0.020), and between ID2 and ID4 (r = 0.460, P = 0.004). However, there was no significant correlation between ID3 and other ID genes.

**Figure 1:**
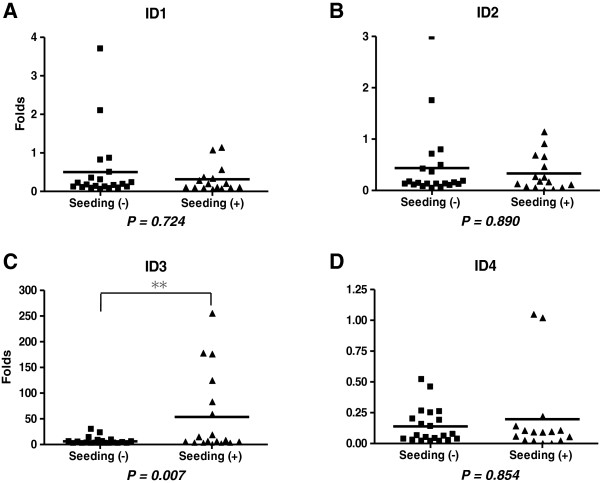
**Expression of ID genes in medulloblastoma.** (**A-D**) RT-qPCR results of ID1, ID2, ID3, and ID4 in 37-39 medulloblastoma tissues dichotomized into seeding (+) and seeding (−) tumors. Horizontal bars represent the average values. ID3 transcript levels were significantly different between the groups.

No significant difference between seeding-negative and seeding-positive groups was observed for ID1, ID2, and ID4 (P = 0.724 for ID1, P = 0.890 for ID2, and P = 0.854 for ID4; Mann–Whitney u-test) (Figure 1A, B, D). In contrast, the seeding-positive group (N = 18) demonstrated significantly higher ID3 transcript levels than the seeding-negative group (N = 21) (53.6 ± 78.3 vs. 6.2 ± 7.6 in folds difference; P = 0.007; Mann–Whitney U test; Figure 1C). ID3 mRNA expression was compared in medulloblastoma cell lines, Daoy and D283. Higher ID3 mRNA expression was observed in D283 than in Daoy (data not shown).

### Knockdown efficiency and specificity of ID3-siRNA and ID3-shRNA

**Figure 2:**
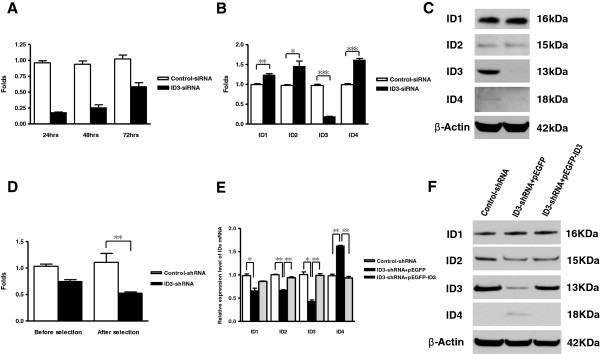
**Knockdown efficiency and specificity of ID3-siRNA and ID3-shRNA.** (**A**) ID3 knockdown greater than 50% for 48 hrs was confirmed after ID3-siRNA transfection to D283 cells. (**B**) ID3 showed a selective reduction of the transcript level after ID3-siRNA treatment. (**C**) Western blot confirmed specific knockdown of ID3 with siRNA. (**D**) D283 cell lines transfected with ID3-shRNA (D283-ID3-shRNA) showed a significant reduction of ID3 mRNA after puromycin selection. (**E**) After transfection with ID3-shRNA, ID1, ID2, and ID3 transcripts levels were decreased and ID4 level was significantly increased. Upon ID3 rescue, ID2 and ID3 transcript levels were restored and ID4 level was also normalized. (**F**) In protein expression, there was a minimal decrease of ID2 expression by ID3-shRNA. The basal ID4 protein expression was negligible in D283 cells. ID4 also showed significant changes in opposite directions of ID3, as indicated in transcript levels.

A stable and specific knockdown of ID3 transcripts of greater than 50% for 48 hrs was confirmed after ID3-siRNA transfection to D283 cells (Figure 2A). ID1, ID2, and ID4 transcripts were not decreased by ID3-knockdown (Figure 2B). Decrease of ID3 protein expression was also confirmed by western blot (Figure 2C).

D283 cell lines transfected with ID3-shRNA (D283-ID3-shRNA) or control-shRNA (D283-control-shRNA) were constructed for in vivo experiments. ID3 transcript levels in RT-qPCR decreased significantly after selection with puromycin (P = 0.003; Student t-test; Figure 2D). Transfection with ID3-shRNA resulted in decrease of ID1, ID2, and ID3 transcripts and increase of ID4 transcript, but only ID3 showed greater than 50% reduction of transcript level compared with the D283-control-shRNA (Figure 2E). Upon rescue of ID3 expression by pEGFP-ID3 vector, both ID2 and ID3 transcript levels were restored and ID4 transcript level was normalized. In protein levels, ID1 expression was not altered either by ID3-shRNA or by ID3 rescue (Figure 2F). ID2 expression was slightly decreased by ID3-shRNA but was restored upon ID3 rescue. ID3 showed dramatic changes of protein expression, closely following the changes of transcript levels. Basal ID4 protein expression was negligible in D283 cells. It showed an increase by ID3-shRNA and a decrease by ID3 rescue, reflecting the changes of transcript levels.

### In vitro assays of D283 cells after transfection with ID3-siRNA

**Figure 3:**
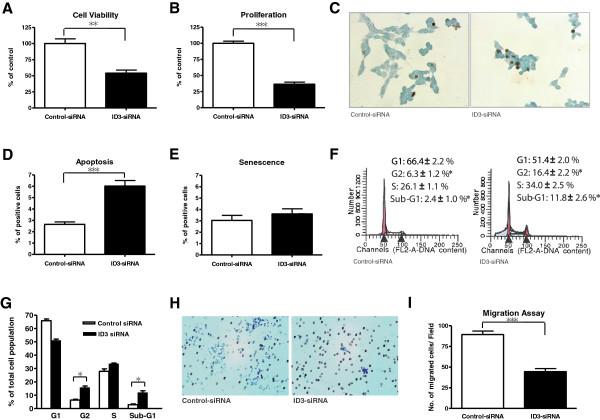
**In vito cellular effects of ID3 knockdown by siRNA in D283 cells.** (**A**) Cellular viability was assessed using CCK assays. There was a significant reduction of cell viability (**B**) Cell proliferation was significantly decreased, assessed using BrdU assays. (**C**) Apoptosis was assessed using TUNEL staining (40×). (**D**) Quantification of TUNEL-positive cells revealed a significant increase of apoptosis after siRNA treatment. (**E**) There was no difference in senescence, assessed using β-gal staining. (**F** and **G**) Cell cycle analysis using FACS showed that G2 phase and sub-G1 fractions were increased after siRNA treatment. Migratory ability was assessed using transwell migration assays. (**H**) Representative field images (100×) and (**I**) the quantification of migrated cells revealed suppression of cell migration by ID3-siRNA. Error bars represent standard deviations.

ID3 knockdown with siRNA significantly decreased cell viability and proliferation of D283 cells. Cell viability after ID3-siRNA transfection was 54.1 ± 4.6% of the controls (P = 0.002; Student t-test; Figure 3A). The percentage of BrdU-incorporating cells after ID3-siRNA transfection was 36.5 ± 3.2% of the controls, indicating decreased proliferation (P < 0.001; Student t-test; Figure 3B).

The impact of ID3 knockdown on cellular apoptosis and senescence was assessed in D283 cells because ID3 knockdown decreased cellular viability. A TUNEL assay revealed a significant increase in apoptosis in ID3-siRNA-transfected cells compared with controls (6.0 ± 0.5% vs. 2.6 ± 0.2%; P < 0.001; Student t-test; Figure 3C and D). No significant difference in SA-ß-gal activity between these groups was observed (3.6 ± 0.4%, vs. 3.0 ± 0.4%; P = 0.174; Student t-test), which indicated that ID3 did not influence cellular senescence (Figure 3E).

Cell cycles in D283 cells transfected with ID3-siRNA and controls were compared. Cell cycle analyses using FACS revealed a significant decrease in the fraction in the G1 phase and an increase in the fractions in G2 and sub-G1 phases after ID3-siRNA transfection compared with controls (G1 phase: 66.4 ± 2.2% vs. 51.4 ± 2.0%, P = 0.002; G2 phase: 6.3 ± 1.2% vs. 16.4 ± 2.2%, P = 0.039; S phase: 26.1 ± 1.1% vs. 34.0 ± 2.5%, P = 0.076; Sub-G1 phase: 2.4 ± 1.0% vs. 11.8 ± 2.6%, P = 0.046; Student t-test; Figure 3F and G). These results indicate an enhancement in G2 arrest and apoptosis after ID3 knockdown. These results are consistent with previous experiments on cellular proliferation and apoptosis.

The in vitro migration ability of D283 cells transfected with ID3-siRNA was compared with that of controls to assess the influence of ID3 gene on medulloblastoma seeding. ID3 knockdown significantly reduced the migration of D283 cells in a transwell migration assay (P < 0.001; Student t-test; Figure 3H and I).

### In vivo migration of D283 cells after ID3-siRNA or ID3-shRNA transfection

A medulloblastoma seeding model was created using nude mice. Diffuse spinal leptomeningeal seeding of tumor cells was confirmed 4 weeks after D283 cell injection into the cisterna magna. The in vivo seeding capability of D283-ID3-shRNA was compared with D283-control-shRNA in this model.

**Figure 4:**
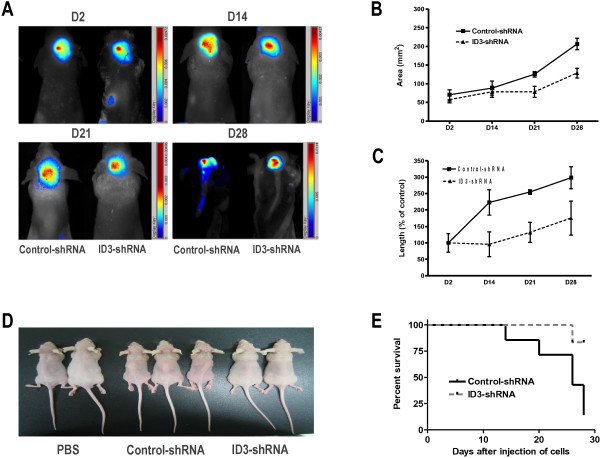
**ID3 knockdown by shRNA and inhibition of tumor seeding in vivo.** (**A**) Representative live in vivo imaging of implanted D283 cells transfected with either control-shRNA or ID3-shRNA for 28 days. Note the migration of signals along the spinal cord of a mouse injected with D283-control-shRNA. (**B**) Serial measurement of tumor-occupied areas in live in vivo imaging. (**C**) Serial measurement of the longitudinal extent of tumors from the cranium to the spinal canal. (**D**) Representative gross photos of mice injected with PBS, D283-controlshRNA, and D283-ID3-shRNA. Mice that received D283-control-shRNA (middle photo) show cachexia and scoliosis, signs of tumor seeding along the spinal cord. (**E**) A Kaplan-Meier survival plot of mice after implantation of D283 cells reveals the increased survival by shRNA. All animals were sacrificed at day 28 for histological examination.

Live in vivo imaging of the mice injected with only PBS (N = 3) or with D283-control-shRNA (N = 7) revealed an enlargement of tumor masses at the injection site for 21 days and seeding along the spinal cord thereafter. In contrast, the mice injected with D283-ID3-shRNA (N = 6) exhibited stable tumor mass sizes at the injection site and no seeding along the spinal cord (Figure 4A). A significant difference in the total areas of optical signal between the groups (D283-control-shRNA vs. D283-ID3-shRNA) was observed (P = 0.028; ANOVA; Figure 4B). The longitudinal length of the optical signals from the cranium to the spinal canal was also significantly different between groups (P < 0.001; ANOVA; Figure 4C).

Grossly, the mice injected with D283-control-shRNA exhibited cachexia, poor hygiene, and scoliosis, which indicated the spinal seeding of tumor cells; mice injected with D283-ID3-shRNA were generally healthy (Figure 4D). A Kaplan-Meier survival curve demonstrated a significant decrease in the survival of mice injected with D283-control-shRNA compared with mice that received D283-ID3-shRNA (P = 0.047; log-rank test; Figure 4E).

**Figure 5:**
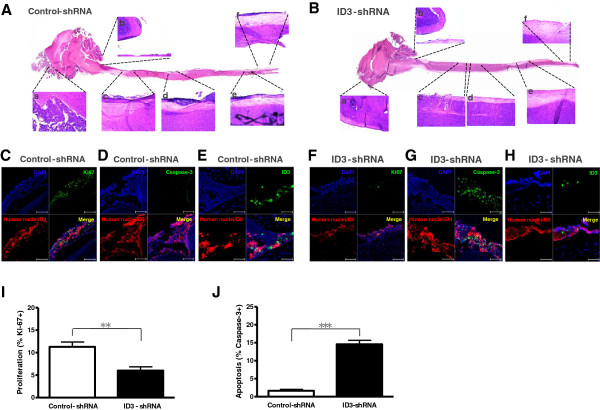
**Histological section of mice for evaluation of tumor seeding.** (**A** and **B**) Representative longitudinal sections of the brains and spinal cords of the mice with D283-control-shRNA or D283-ID3-shRNA (hematoxylin & eosin stain) (1.25×). Inlet figures denote (a) subfrontal area, (b) craniocervical junction (cistern magna) where the tumor cells were first implanted, (c) upper thoracic spinal cord, (d) midthoracic spinal cord, (e) lumbar spinal cord, and (f) conus medullaris (100×). (**C** and **F**) Immunofluorescence (IF) staining with anti-Ki-67, a proliferation marker, on the specimen. (**D** and **G**) IF staining with anti-caspase-3, an apoptosis marker. Scale bars represent100 μm. (**E** and **H**) IF staining with anti-ID3. (**I** and **J**) Comparison of the proportions of Ki-67^+^ cells and caspase-3^+^ cells.

Postmortem histological examination revealed huge tumor masses at the injection site and diffuse and thick leptomeningeal seeding of tumor cells in mice injected with D283-control-shRNA, but tumor cells were scarcely observed in mice that received D283-ID3-shRNA (Figure 5A and B). Immunofluorescence staining revealed that abundant Ki-67^+^ tumor cells were observed in control mice, but mice injected with D283-ID3-shRNA had few Ki-67^+^ tumor cells (11.29 ± 3.67% in D283-control-shRNA vs. 6.00 ± 2.89% in D283-ID3-shRNA; P = 0.004; Student t-test; Figure 5C, F, I). On the contrary, abundant caspase-3-expressing tumor cells were observed in mice injected with D283-ID3-shRNA (1.67 ± 1.23% in D283-control-shRNA vs. 14.58 ± 3.80% in D283-ID3-shRNA; P < 0.001; Student t-test; Figure 5D, G, J). ID3 expression was effectively suppressed in mice that received D283-ID3-shRNA (Figure 5E and H). No difference of ID2 expression between the groups was observed and anti-ID4 fluorescence signal was too weak to detect in both groups (data not shown).

### Expression of cellular invasion and migration genes after ID3-siRNA transfection in D283 cells

**Figure 6:**
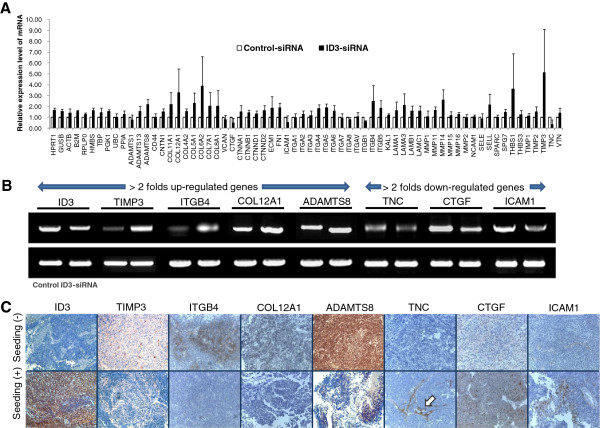
**mRNA mini-array to uncover ID3-related genes.** (**A**) Expression of 66 genes related to cellular invasion and metastasis after ID3 knockdown using siRNA in D283 cells. (**B**) RT-PCR of ID3 and the 7 genes with considerable initial expression levels and expression level changes of greater than 2-fold. (**C**) Representative images of immunohistochemistry of ID3 and 7 proteins in seeding-negative and seeding- positive medulloblastomas (200×). Note that TNC is mainly expressed in the tumor stroma (arrow) than tumor cell clusters where ID3 immunoreactivity is observed.

Sixty-six cellular invasion and migration genes were detectable in D283 cells using the mRNA miniarray. Thirteen genes [(TIMP3, collagen type VI alpha 2 (COL6A2), thrombospondin 1 (THBS1), COL12A1, matrix metallopeptidase 14 (MMP14), ITGB4, collagen type V alpha 1 (COL5A1), collagen type I alpha 1 (COL1A1), ADAMTS8, selectin L (SELL), laminin alpha 3 (LAMA3), collagen type VII alpha 1 (COL7A1), collagen type VIII alpha 1 (COL8A1)] were upregulated, and 3 genes (TNC, CTGF, ICAM1) were downregulated more than 2-fold after ID3 knockdown in D283 cells in vitro (Figure 6A). Stably transcribed genes were selected by discarding genes without amplification peaks at 35 cycles in RT-qPCR processes. Four upregulated genes (TIMP3, ITGB4, COL12A1, ADAMTS8) and 3 downregulated genes (TNC, CTGF, ICAM1) were associated with ID3 knockdown. These results were confirmed using RT-PCR (Figure 6B). Immunohistochemistry of ID3, TIMP3, ITGB4, COL12A1, ADAMTS8, TNC, CTGF, and ICAM1 in human medulloblastoma tissues demonstrated different protein expression patterns according to the seeding status of the disease. A higher expression of TIMP3, ITGB4, COL12A1, and ADAMTS8 was observed in the seeding-negative group, and a higher expression of ID3 and CTGF was observed in the seeding-positive tumors. There were slightly stronger expression of TNC and ICAM1 in the seeding-positive tumors, but the immunopositive areas were restricted to tumor stroma rather than tumor cell clusters where the majority of ID3-immunoreactivity was found (Figure 6C).

### Molecular subgroup of tumors

**Figure 7:**
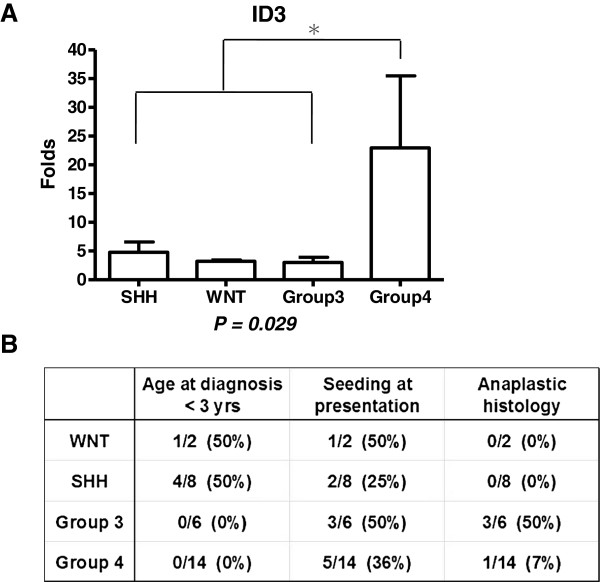
**Molecular subgroup of medulloblastoma and ID3.** (**A**) ID3 expression assessed with RT-qPCR according to the medulloblastoma subgroups (N = 30). (**B**) Clinical characteristics (young age at diagnosis < 3 yrs, seeding at presentation, and anaplastic histology) of patients in each subgroup.

The molecular subgroups of 30 tumors were identified: WNT subgroup (2 tumors), SHH subgroup (8 tumors), Group 3 (6 tumors), and Group 4 (14 tumors). ID3 transcript levels in RT-qPCR of these subgroups were compared. Group 4 tumors showed significantly higher levels of ID3 mRNA than other subgroups (P = 0.029; Mann–Whitney U test; Figure 7A). Important clinical profiles of the patients in each subgroup were summarized in Figure 7B. Age at diagnosis less than 3 yrs was mainly observed in SHH subgroup and Group 3 showed highest rate of anaplastic histology. In Group 4, there was no patient with young-age-onset less than 3 yrs and only 1 patient had an anaplastic medulloblastoma.

### Survival of medulloblastoma patients according to ID3 expression

**Figure 8:**
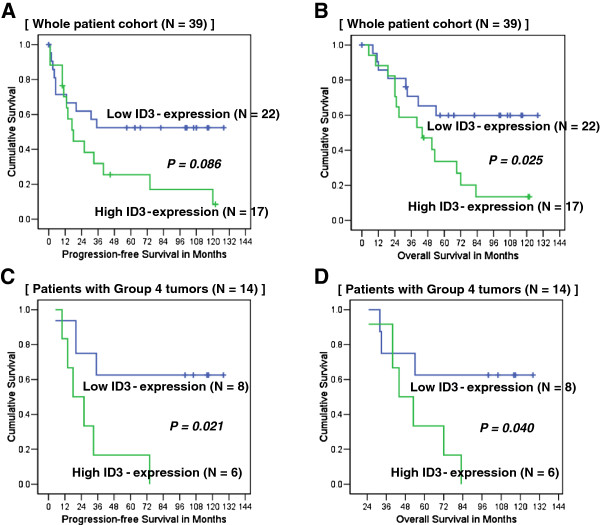
**Patient survival according to ID3 expression levels.** (**A** and **B**) Kaplan-Meier survival curves for progression-free survival (PFS) and overall survival (OS) in 39 patients with medulloblastoma. Fold changes greater than 6.007 in RT-qPCR was considered as high ID3-expression. (**C** and **D**) Survival plots for PFS and OS in 14 patients who had Group 4 medulloblastomas.

The survival of patients with medulloblastoma in whom ID3 expression levels were assessed using RT-qPCR was analyzed. During the follow-up, 22 patients (56%) expired and 17 patients (44%) were censored. Because of the wide range of ID3 expression levels in patients with tumor seeding, ID3 expression levels were dichotomized into high and low expression levels (> 6.007-fold difference vs. ≤ 6.007-fold difference, respectively) relative to the expression level of normal cerebellum. A total of 17 patients were placed in the high ID3-expression group, and 22 patients exhibited low ID3 expression. The clinical characteristics of each group are summarized in Table 1. Only seeding at presentation were significantly more frequent in the high ID3-expression group than in the low ID3-expression group (P = 0.01), all the other prognostic factors did not show any statistical difference between the high and low ID3 expression groups. Kaplan-Meier curves demonstrated that the high ID3-expression group had marginally significantly shorter PFS than the low ID3-expression group (P = 0.086; log-rank test; Figure 8A). The high ID3- expression group also had significantly shorter OS than the low ID3-expression group (P = 0.025; log-rank test; Figure 8B). Multivariate analyses revealed that high-ID3 expression was an independent risk factor of death in patients with medulloblastoma after the adjustment of major prognostic factors (P = 0.034, aHR = 3.840, 95% CI = 1.107 - 13.312). The risk for progression of medulloblastoma by the high expression of ID3 was 2.137 times (95% CI: = 0.684 - 6.676), which was not statistically significant after the adjustment (P = 0.192) (Table 2). Age younger than 3 yrs old at the diagnosis, seeding at presentation, anaplastic histology were statistically significant risk factors for both outcomes, however, residual tumor larger than 1.5 cm^2^ was not significant after the adjustment.

**Table 1:** Comparison of clinical characteristics between high ID3-expressing and low ID3-expressing groups

	**ID3-expressing group**			
**Characteristics**	**Low (N = 22)**	**%**	**High (N = 17)**	**%**
Mean age (± SD; yrs)	3.63 (± 3.42)		4.70 (± 4.06)	
Sex (male)	15	68.2	12	70.6
Age at diagnosis (<3 yrs)	4	18.2	4	23.5
Residual tumor (> 1.5 cm^2^)	2	9.1	1	5.9
Seeding at presentation	6	27.3	12	70.6
Anaplastic histology	4	18.2	0	0

SD = standard deviation.

**Table 2:** Relative risks for shorter progression-free survival (PFS) and overall survival (OS) estimated with Cox proportional hazard models

	**Crude HR**	**95% Hazard ratio confidence interval**		**Adjusted HR**	**95% Hazard ratio confidence interval**	
PFS						
Sex	1.383	0.571	3.349	0.745	0.276	2.012
Age at diagnosis (<3 yrs)	2.558	0.989	6.617	4.612	1.527	13.927
Residual tumor (> 1.5 cm^2^)	27.303	4.299	173.409	5.990	0.681	52.719
Seeding at presentation	3.871	1.663	9.008	3.546	1.142	11.014
Anaplastic histology	3.254	1.086	9.748	8.229	1.465	46.242
High ID3 expression	2.018	0.887	4.595	2.137	0.684	6.676
OS						
Sex	2.043	0.747	5.585	1.286	0.428	3.863
Age at diagnosis (<3 yrs)	2.195	0.780	6.179	4.323	1.249	14.959
Residual tumor (> 1.5 cm^2^)	4.374	1.225	15.619	0.680	0.120	3.834
Seeding at presentation	4.734	1.881	11.914	3.373	1.113	10.222
Anaplastic histology	3.200	1.056	9.691	13.235	2.058	85.090
High ID3 expression	2.609	1.090	6.249	3.840	1.107	13.312

In the patients with Group 4 tumors (N = 14), detailed analysis of risk factor was not indicated because of the small number of patients. Nonetheless, considering that age younger than 3 yrs and anaplastic histology were far less represented in this subgroup, high ID3 expression may have more impact than the whole patient cohort. High ID3-expression group (N = 6) had significantly shorter PFS and OS than the low ID3-expression group (P = 0.021 and P = 0.040, respectively; log-rank test; Figure 8C and D).

## Discussion

ID genes are known as transcriptional repressors and have important roles in developmental processes. There are four ID gene homologues, ID1, ID2, ID3, and ID4 in human and other vertebrates [19]. The functions of ID genes must be redundant and depend on the cellular context to some degree. Knockout of either ID1 or ID3 alone in mice generated apparently normal phenotypes [31]. There are also functional interactions between ID genes. ID3-shRNA used for this experiment showed a significant on-target effect on ID4 (down-regulation) and a minimal influence on ID2 expression. It is known that ID3 can down-regulate ID4 in a specific cellular context [32]. Furthermore, in medulloblastoma tissues and cell lines examined (D283 and Daoy), basal ID4 transcript level and protein expression was negligible compared with those of ID3. Therefore, we focused on the functional role of ID3 in medulloblastoma.

Overexpression of ID genes is widely reported in human cancers, including cancers of gastrointestinal tract, breast, prostate, endometrium, cervix, and thyroid, to name a few [19]. Their expression is further regarded as poor prognostic factor in some of the cancers [33]. ID1, ID2, and ID3 are known to regulate cell fate determination and to maintain undifferentiated states [19]. Therefore, they can keep tumor cells in stem-cell-like states or cause dedifferentiation (anaplasia). Actually, ID1 has been proposed as a marker of glioma-initiating cells [34]. ID genes can promote cell proliferation and prevent apoptosis, the two key properties of cancer cells. Knockdown experiments of ID genes in various cancer cell lines showed decreased proliferation and enhanced apoptosis in vitro. One of the most crucial actions of ID genes is their involvement in cell migration, invasion, and angiogenesis which makes ID genes promote metastasis. Knockdown of ID1 and ID3 inhibited metastatic potentials of esophageal and pancreatic cancers in vitro and in vivo [25,35]. Inhibition of metastasis-suppressing genes and promotion of epithelial-mesenchymal transition have been proposed as the mechanisms of action [36,37].

Medulloblastoma is characterized by high rates of tumor seeding through the neuraxis which occurs at both early and terminal stages of the disease. Tumor seeding at presentation is a strong predictor of poor outcome in medulloblastoma [8]. Tumor seeding also affects long-term quality of life of survivors, because many clinical protocols on medulloblastoma mandate more intensified treatment for a disseminated disease than a localized tumor. Recently, genomic characteristics of medulloblastoma are being unveiled and the molecular classification demonstrates that the disease consists of at least 4 distinct subgroups [12,13]. In this context, key genetic players and molecular mechanisms of medulloblastoma seeding are arousing much interest [17].

In the present study, we explored whether ID genes are associated with medulloblastoma seeding. The transcript level of ID3 was much higher in medulloblastomas than in normal cerebellum, and was also independent of other ID genes. Furthermore, ID3 transcripts were differentially elevated in seeding-positive medulloblastomas than in seeding-negative tumors. Considering the general functions of ID genes in many human cancers, we postulated that ID3 may be a potential player in medulloblastoma seeding.

Knockdown of ID3 in the medulloblastoma cell line resulted in decrease of cell viability and proliferation, enhanced apoptosis, and suppressed migratory activities in vitro. A study showed that ID1 and ID3 facilitated sustained proliferation during the early stages of metastatic colonization of breast carcinoma [26]. This finding indicates that not only increased migration/ invasion capability but also enhanced survival at the remote sites may contribute to the metastasis-promoting action of ID genes. Therefore, the association of ID3 with medulloblastoma seeding may depend on these pleiotropic functions of ID3 gene. In vivo study of ours reinforced this concept. In the animal seeding model of medulloblastoma, stable knockdown of ID3 in injected tumor cells lead to a decreased extent of tumor seeding and prolonged survival of mice. The tumor masses formed in the primary injection were also much smaller than controls. On histological examination, a scattered thin layer of tumor cells was observed on the spinal leptomeninges, but the tumor cells lacked proliferative activities and showed high proportion of apoptosis. From these findings, ID3 could be viewed better as an indicator of disease aggressiveness rather than simply as a metastasis-promoting factor.

Many genes must be involved in the multiple actions of ID3 in establishment of metastasis. Through a small array system and tumor cell line, we found several candidate genes of ID3 targets. The most intriguing genes may be TNC and CTGF that showed downregulation after ID3 knockdown. TNC is a candidate oncongene responsible for disease progression of ependymomas [38]. There is a report that TNC protein and its partner integrins mediate adhesion of medulloblastoma cells to leptomeninges and facilitate tumor seeding [39]. However, in our study, the protein expression of TNC was generally limited to the tumor stroma, apart from tumor cell clusters where most of ID3 immunoreactivity was observed. Therefore, the cross-talk between tumor cells and microenvironment needs further elucidation. CTGF is known to be a member of Musachi1-associated gene network which is highly expressed in aggressive medulloblastoma [40]. Although the mRNA expression of thrombospondin-1 (THBS1) was not augmented in D283 cells in our experiment, THBS1 was upregulated after silencing of ID3. A previous study demonstrated that downregulation of THBS1 was strongly associated with MYC-driven metastatic phenotype of medulloblastoma [17].

In the RT-qPCR results of ID genes (Figure 1), ID3 transcript levels were not uniformly elevated in the seeding-positive group, but only a small number of tumors showed high expression of ID3. This finding may indicate that medulloblastomas have diverse seeding mechanisms and ID3 may represent one of the machinery that acts in a limited group of patients. In the prognostic analyses using the patients’ clinical data, high ID3-expression was an independent negative prognostic factor, but it was associated only with OS, without significantly affecting PFS. Traditional risk factors such as young age at diagnosis (< 3 yrs), seeding at presentation, and anaplastic histology all significantly influenced both PFS and OS in the whole patient cohort. However, it should be noted that the confidence intervals of hazard ratios are rather wide, indicating that they are based on a small number of patients and events.

It is well established that medulloblastomas are heterogeneous tumors in which molecular classification is possible. Therefore, we obtained information on the subgroup allocations and compared ID3 expression between the subgroups. Although the allocated numbers are small in each subgroup, their clinical characteristics were consistent with the published data [12,13]: young age at diagnosis (< 3 yrs) in SHH subgroup, high proportions of seeding at presentation and anaplastic histology in Group 3, and relatively low proportions of young age at diagnosis (no patient) and anaplastic histology (only 1 patient) in Group 4. Interestingly, Group 4 medulloblastomas showed significantly higher ID3 expression than other subgroups. This finding may have intriguing implications. In the current molecular classification, Group 3 tumors are associated with anaplastic histology, MYC amplification, metastatic phenotype, and dismal prognosis. Experimentally, high MYC expression induces metastatic tumors in orthotopic medulloblastoma models [17].

Group 4 medulloblastomas have a higher proportion of seeding at presentation than WNT and SHH subgroups, but MYC amplification and anaplasia are seldom found in the subgroup [13]. We can postulate that these medulloblastoma subgroups have distinct mechanisms of tumor seeding driven by different genes. Therefore, ID3 may represent the metastatic/ aggressive phenotype of Group 4 medulloblastomas that lack MYC amplification. Survival analyses of patients with Group 4 tumors reinforced this assumption. In Group 4 tumors, high ID3 expression may have greater prognostic impact because these tumors have higher ID3 expression than other subgroups, and because young age at diagnosis and anaplastic histology, the two robust risk factors were virtually excluded from this group (0 and 1 patient each). Despite the small number of patients with Group 4 tumors (N = 14), high ID3-expression was more represented as a poor prognostic factor in this subgroup, significantly affecting both PFS and OS.

## Conclusion

High ID3 expression was associated with medulloblastoma seeding at presentation, but not all tumors with seeding had high ID3 expression. Silencing of ID3 in D283 cell line decreased proliferation, increased apoptosis, and suppressed migration in vitro. In vivo knockdown experiment demonstrated that ID3 not only increased migration capability, but also enhanced survival at the metastatic loci of medulloblastoma cells. In survival analysis of the patients, high ID3 expressions emerged as a poor prognostic factor, especially in patients with Group 4 medulloblastomas. ID3 may represent the metastatic/aggressive phenotype of Group 4 medulloblastomas.

## Abbreviations

CSF: Cerebrospinal fluid; ID: Inhibitor of differentiation; bHLH: Basic helix-loop-helix; EMEM: Minimum Essential Medium Eagle; DMEM: Dulbecco’s Modified Eagle’s Medium; FBS: Fetal bovine serum; PCR: Polymerase chain reaction; TIMP3: Tissue inhibitor of metalloproteinase 3; ITBG4: Integrin beta 4; COL12A1: Collagen type XII alpha1; ADAMTS8: ADAM metallopeptidase with thrombospondin type 1 motif 8; TNC: Tenascin C; CTGF: Connective tissue growth factor; ICAM1: Intercellular adhesion molecule 1; COL6A2: Collagen type VI alpha 2; MMP14: Matrix metallopeptidase 14; COL5A1: Collagen type V alpha 1; COL1A1: Collagen type I alpha 1; SELL: Selectin L; LAMA3: Laminin alpha 3; COL7A1: Collagen type VII alpha 1; COL8A1: Collagen type VIII alpha 1; ANOVA: Analysis of variance; PFS: Progression-free survival; OS: Overall survival.

## Competing interests

The authors report no competing interests.

## Authors’ contributions

JHP initiated the experiment and wrote the manuscript. SAC and S-HL did in vitro and in vivo experiments. JL is responsible for the statistical analyses of the study. K-CW discussed the concept and critically reviewed the manuscript. S-HP reviewed the pathological slides of both surgical specimen and experiments. S-KK is the corresponding authors and produced the whole article. All authors read and approved the final manuscript.

## Pre-publication history

The pre-publication history for this paper can be accessed here:

http://www.biomedcentral.com/1471-2407/13/291/prepub

## Supplementary Material

Additional file 1: Table S1 Primer sequences and parameters for reverse transcriptase polymerase chain reaction. Click here for file
